# The Narrow Meatus—How Produced

**Published:** 1877-07

**Authors:** John Thad. Johnson

**Affiliations:** Atlanta, Ga.; Prof. of Anatomy and Lecturer on Venereal Diseases in the Atlanta Medical College


### THE NARROW MEATUS—HOW PRODUCED.

By JNO. THAD. JOHNSON, M.D.,
Prof. of Anatomy and Lecturer on Venereal Diseases in the Atlanta Medical College.

(*An Essay read before the Atlanta Academy of Medicine.*)

In the January number of the ATLANTA MEDICAL AND SURGICAL JOURNAL, I presented a short article on the Narrow Meatus as a Cause of the Stricture of the Urethra I will here speak briefly of the mechanism of formation of this abnormally small opening.

Surprise, or doubt, is occasionally expressed by physicians at the frequency of this malformation; they seeming to regard this opening as determined by a fixed law of nature, from which variations seldom occur. Almost every portion of the anatomy of the body is subject to malformation of deficiency or redundancy. Certain structures suffer in this way more frequently than others. This is true of a class known as middle line deformities, and of the mucous outlets of the body. A deformity very frequently met with is the deficiency on the under surface of the anterior portion of the male urethra—a hypospadias of limited extent. This seems to be owing in great part to a deficiency of the mucous covering of the glans.

On the other hand, we find the reverse condition obtaining at times; the mucous membrane being prolonged *too far* forward, leaving the opening of the urethra too little, instead of too much exposed, as in the condition just spoken of. If any one will carefully observe a small meatus of this type, it will be found that the abnormality is dependent on this mucous membrane occupying the inferior commissure of the termination of the urethra. By inserting an instrument—say a probe or small bougie—within the opening, and pressing it forward against the membrane, the condition is readily demonstrated. I have seen several patients whose meatus would barely admit a No. 1 bougie, in whom the deformity depended entirely on this process of membrane, the urethra itself being perfect. As a rule, though, even when the deformity is present, the membrane does not occupy so much of the opening as in the above instances.

In the normal meatus even, the extreme inferior commissure is occupied by this membrane, or fourchette. It requires but a very small mistake for nature to overstep the normal size, and extend the mucous covering higher up the opening. This seems very like the formation and the variations of that partial covering of the genital canal in the female, the hymen. This membrane occupies almost invariably the lower commissure of the vagina, is continued from the general mucous covering of the part, and, like the fold stretched across the termination of the male urethra, is subject to much variation as to the height to which it ascends on the opening.

It will also be observed that this mucous membrane, in the male, is a continuation, or expansion, directly from the frænum—the mucous layer of the prepuce being gathered into the ridge, or fold, known as the frænum, and it in turn expanding at the lower commissure of the meatus.

This congenitally small meatus, then, as above described, is not a true stricture of the urethra, in the sense expressed by that term; that is, *its structure is different, its mode of formation is different;* the one is a normal tissue, very slightly deformed or prolonged; the other is a new structure, a pathological formation.

The question has been asked, why may not a deep organic structure exist congenitally as readily as the small meatus? The above paragraph I give as the explanation. There may be found occasional cases in which the anterior portion of the urethra proper, the channel through the glans penis, is involved in a congenital constriction, but they are comparatively rare. They do not constitute that typical variety so often met with, and whose mode of production accounts for the many differences in the size of the meatus as compared with the calibre of the urethra, even within the limits of health.

I do not propose to speak here of diseases or symptoms depending on the malformation in question, further than allude to those disorders as set forth in the article in the Journal, already referred to. Further observation has confirmed those views. It led me also to investigate the fact of there being so little relation between the size of the urethra and of that of the meatus.

This small meatus should not be confounded with an organic stricture, not congenital, which is so frequently found within one-quarter or one-half an inch of the meatus—and yet such confusion is made under the term of constricted meatus.

When the meatus is properly enlarged by the knife, and after-treatment conducted properly, there is but little danger of its returning to the former size. But when the true stricture just behind the meatus is divided, there will be subsequent recontraction, as occurs in strictures of other parts of the canal.

